# LFD-YOLO: a lightweight fall detection network with enhanced feature extraction and fusion

**DOI:** 10.1038/s41598-025-89214-7

**Published:** 2025-02-11

**Authors:** Heqing Wang, Sheng Xu, Yuandian Chen, Chengyue Su

**Affiliations:** 1 School of Physics and Optoelectronic Engineering, Guangdong University of Technology, Guangzhou, 510006 Guangdong China; 2 School of Advanced Manufacturing, Guangdong University of Technology, Jieyang, 515200 Guangdong China

**Keywords:** Object detection, Fall detection, Feature extraction, Feature fusion, Inner-WIoU loss, Computational science, Computer science

## Abstract

Falls are one of the significant safety hazards for the elderly. Current object detection models for fall detection often suffer from high computational complexity, limiting their deployment on resource-constrained edge devices. Although lightweight models can reduce computational requirements, they typically compromise detection accuracy. To address these challenges, and considering the more lightweight architecture of YOLOv5 compared to other YOLO series models such as YOLOv8, we propose a lightweight fall detection model based on YOLOv5, named Lightweight Fall Detection YOLO (LFD-YOLO). Our method introduces a novel lightweight feature extraction module, Cross Split RepGhost (CSRG), which reduces information loss during feature map transmission. We also integrate an Efficient Multi-scale Attention (EMA) to enhance focus on the human pose. Moreover, we propose a Weighted Fusion Pyramid Network (WFPN) and utilize Group Shuffle Convolutions (GSConv) to reduce the model’s computational complexity and improve the efficiency of multi-scale feature fusion. Additionally, we design an Inner Weighted Intersection over Union (Inner-WIoU) loss to accelerate model convergence and enhance generalization. We construct a Person Fall Detection Dataset (PFDD) dataset covering diverse scenarios. Experimental results on the PFDD and the publicly available Falling Posture Image Dataset (FPID) datasets show that, compared to YOLOv5s, LFD-YOLO improves mAP0.5 by 1.5% and 1.7%, respectively, while reducing the number of parameters and calculations by 19.2% and 21.3%. Furthermore, compared to YOLOv8s, LFD-YOLO reduces the number of parameters and calculations by 48.6% and 56.1%, respectively, while improving mAP0.5 by 0.3% and 0.5%. These results demonstrate that LFD-YOLO achieves higher detection accuracy and lower computational complexity, making it well-suited for fall detection tasks.

## Introduction

In recent years, the global trend of population aging has become increasingly evident. As life expectancy rises and fertility rates decline, the proportion of elderly individuals aged 60 and above continues to increase. With advancing age, older adults’ mobility and balance abilities gradually decline, leading to an increased risk of falls. Therefore, research into fall detection technology for the elderly has become critically important^1^. Such technology can detect and respond to falls in real time, reducing fall-related injuries and providing reliable safety assurance for older adults. This research holds significant social and practical value.

Fall detection technology has garnered significant attention due to its broad application prospects and practical value. Researchers have explored various methods for fall detection^2,3^. Computer vision-based fall detection technologies can be divided into human pose estimation and object detection algorithms. These methods capture real-time images through cameras and utilize deep learning models to extract human features for fall detection. In fall detection research based on human pose estimation algorithms, Anitha et al.^4^ employed the AlphaPose algorithm to extract skeletal motion features and analyzed human poses to detect falls. Gao et al.^5^ utilized the OpenPose algorithm to capture human posture information and combined it with an improved MobileNetV2 algorithm for fall detection. Wei et al.^6^ integrated the modified BlazePose with Long Short-Term Memory (LSTM) network to capture human actions and classify them to detect falls. Liang et al.^7^ proposed the Relative Position Encoding Pose Estimation (RPEpose) algorithm to extract keypoint information. They built a Cross-Joint Attention Graph Convolutional Network (xJ-GCN) to classify human behavior based on keypoint relations. Liu et al.^8^ utilized a Spatio-Temporal Adaptive Graph Convolutional Network (ST-AGCN) to extract human motion features for fall identification. Amsaprabhaa et al.^9^ combined a one-dimensional Convolutional Neural Network (1D-CNN) with a Spatio-Temporal Graph Convolutional Network (STGCN) to capture human motion features and construct a classification model for fall detection. While human pose estimation algorithms can recognize changes in body posture, they are prone to higher computational errors and costs due to the multiple computational stages involved in keypoint extraction and analysis.

In contrast, object detection algorithms^10^ directly detect fall events by extracting human features, simplifying the recognition process, and exhibiting lower computational errors and network complexity. Object detection algorithms are classified into two categories: two-stage and one-stage. Although two-stage algorithms, such as SPP-Net^11^, Faster R-CNN^12^, and R-FCN^13^, offer higher detection accuracy, their complex network structures and slower inference speeds limit their application in real-time fall detection. In comparison, one-stage algorithms, especially those in the YOLO series^14^, adopt an end-to-end behavior detection approach characterized by speed, efficiency, and flexibility. In object detection algorithms, detection accuracy and computational complexity are key performance metrics. While YOLO-based algorithms applied to fall detection achieve high accuracy, they also demand significant computational resources, which limits their deployment on resource-constrained edge devices. Furthermore, the model’s detection speed decreases, leading to an inability to respond to falls in real time. To address this, lightweight YOLO algorithms reduce computational resource demands by simplifying the model structure or incorporating lightweight modules. However, this often comes at the cost of detection accuracy, leading to false positives and missed detections, compromising the model’s overall reliability and stability in fall detection tasks.

To address the limitations of the YOLO algorithm in fall detection tasks and balance detection accuracy with computational complexity, this research proposes a lightweight fall detection model based on YOLOv5, named LFD-YOLO (Lightweight Fall Detection YOLO). Compared to other models in the YOLO series, such as YOLOv8, the YOLOv5 structure is more lightweight. First, we introduce a Cross Split RepGhost (CSRG) lightweight feature extraction module, which reduces information loss during transmission and enhances feature extraction capabilities. Additionally, we design an improved Weighted Feature Pyramid Network (WFPN) and utilize Group Shuffle Convolutions (GSConv) to replace standard convolutions. This reduces computational complexity while enhancing the efficiency of multi-scale feature fusion. Furthermore, we incorporate an Efficient Multi-Scale Attention (EMA) to enhance focus on human pose details through parallel branch computations. Lastly, we design an Inner Weighted Intersection over Union (Inner-WIoU) loss function to improve the accuracy of human localization. The main contributions of this research are as follows:

- Designed a lightweight fall detection model, LFD-YOLO, based on YOLOv5, which integrates the CSRG lightweight feature extraction module, the improved WFPN structure, the EMA attention mechanism, and the Inner-WIoU loss function. These improvements enhance detection accuracy while maintaining the model’s lightweight structure and real-time processing capability.
- Proposed a CSRG lightweight feature extraction module and introduce an EMA attention mechanism to reduce feature information loss during propagation, capturing more detailed human pose information and enhancing feature representation capabilities. The feature fusion network is optimized to the WFPN structure, and GSConv replace traditional standard convolutions. These improvements reduce the model’s computational complexity while enhancing the information representation ability of multi-scale feature maps.
- Designed an Inner-WIoU loss function to improve the adaptability of bounding boxes to human pose at different scales, accelerate model convergence, and enhance localization accuracy and generalization ability.
- Constructed a Person Fall Detection Dataset (PFDD) dataset, which includes images captured from multiple angles, under different lighting conditions, and with partial occlusion of the human body. The diversity of the images ensures the dataset’s authenticity, thereby enhancing the robustness and generalization capabilities of the model. Experimental results based on the publicly available Falling Posture Image Dataset (FPID) and PFDD datasets demonstrate that, compared to state-of-the-art model, LFD-YOLO improved the mAP0.5 metric by 0.5% and 0.3%, respectively.

## Related work

### Lightweight network for fall detection

Fall detection algorithms are typically deployed on resource-constrained edge devices, requiring real-time detection of falls. Therefore, lightweight model design is critical for efficient performance. Current research primarily focuses on lightweight module design and network structure simplification. Luo et al.^15^ reduced the parameters by replacing standard convolutions and C3 modules in YOLOv5 with Ghost convolutions and C3GhostV2 modules. Xi et al.^16^ integrated GSConv and the lightweight GDCN module into the feature fusion network of YOLOv5, reducing the overall model size. Zheng et al.^17^ replaced C2f modules in YOLOv8 with the more efficient FasterNet modules, introducing deformable convolutions to replace standard convolutions, thus lowering computational complexity. Dai et al.^18^ designed the lightweight detection head based on YOLOv5, removing redundant channels to reduce computational costs. Wang et al.^19^ replaced the feature extraction network of YOLOv5 with the lightweight ShuffleNetV2, simplifying the network structure.

Although these lightweight improvements reduce the computational complexity of the model, they are often accompanied by a decrease in detection accuracy, especially in fall detection scenarios involving human occlusion or low-light conditions. To address these challenges, we proposes the CSRG feature extraction module to replace the C3 module in YOLOv5. This modification reduces computational burden while enhancing feature representation. GSConv is also introduced to make it further lightweight. Additionally, the EMA attention mechanism and Inner-WIoU loss function are integrated to enhance attention to human posture, improving the detection accuracy and generalization ability of the model.

### Multi-scale feature extraction and fusion for fall detection

In fall detection tasks, multi-scale feature extraction and fusion enable the model to simultaneously handle low-level detailed features and high-level semantic information, enhancing the ability to represent features across different scales. Current research primarily focuses on improving network structures and incorporating attention mechanisms. Fan et al.^20^ designed the TModule feature extraction module, introducing recursive gated non-local convolution (gnconv) to better capture contextual information from multi-scale feature maps. Qin et al.^21^ replaced the C2f module in YOLOv8 with the improved C2Dv3 module and introduced the DyHead detection head, capturing more complex details. Zhang et al.^22^ proposed a Bi-directional Cascaded Feature Pyramid Network (BiC-FPN), optimizing the feature fusion network in YOLOv5 and improving the fusion efficiency of multi-scale feature maps. Zhao et al.^23^ designed a MJPANet feature fusion network, incorporating the Convolutional Block Attention Module (CBAM) to reduce information loss during feature map transmission. Chen et al.^24^ integrated the Efficient Channel Attention (ECA) module and average pooling layer into the Spatial Pyramid Pooling (SPP) module, enhancing the feature extraction of multi-scale fall postures.

Although existing multi-scale feature extraction and fusion methods improve the model’s detection accuracy, different scale feature maps may contain redundant or repetitive information, which increases computational cost. This limits the model’s deployment on resource-constrained edge devices and reduces its inference speed. Furthermore, due to the varying information across different scales, information loss during feature processing may degrade the model’s detection performance. To address these challenges, this research proposes the lightweight CSRG feature extraction module, incorporating the Split RepGhost (SRG) bottleneck to minimize information loss during feature extraction and provide richer feature map information. Additionally, we introduce the WFPN structure, which dynamically adjusts the contribution of each layer’s feature map to enhance the expressive power of multi-scale feature map information.

### YOLOv5 network structure

As a one-stage object detection algorithm, the YOLO series is renowned for its high accuracy, real-time performance, and ease of deployment, making it widely applicable across various domains^25^. Among these, YOLOv5 is the most mature and stable version. Compared to other versions, such as YOLOv8, YOLOv5 features a more lightweight network structure, enabling efficient detection performance even on resource-constrained embedded devices. This makes it particularly well-suited for real-time fall detection tasks. YOLOv5 offers five model variants-n, s, m, l, and x-based on different network depths and widths. For real-time fall detection, we selects YOLOv5s as the baseline model.

**Fig. 1:**
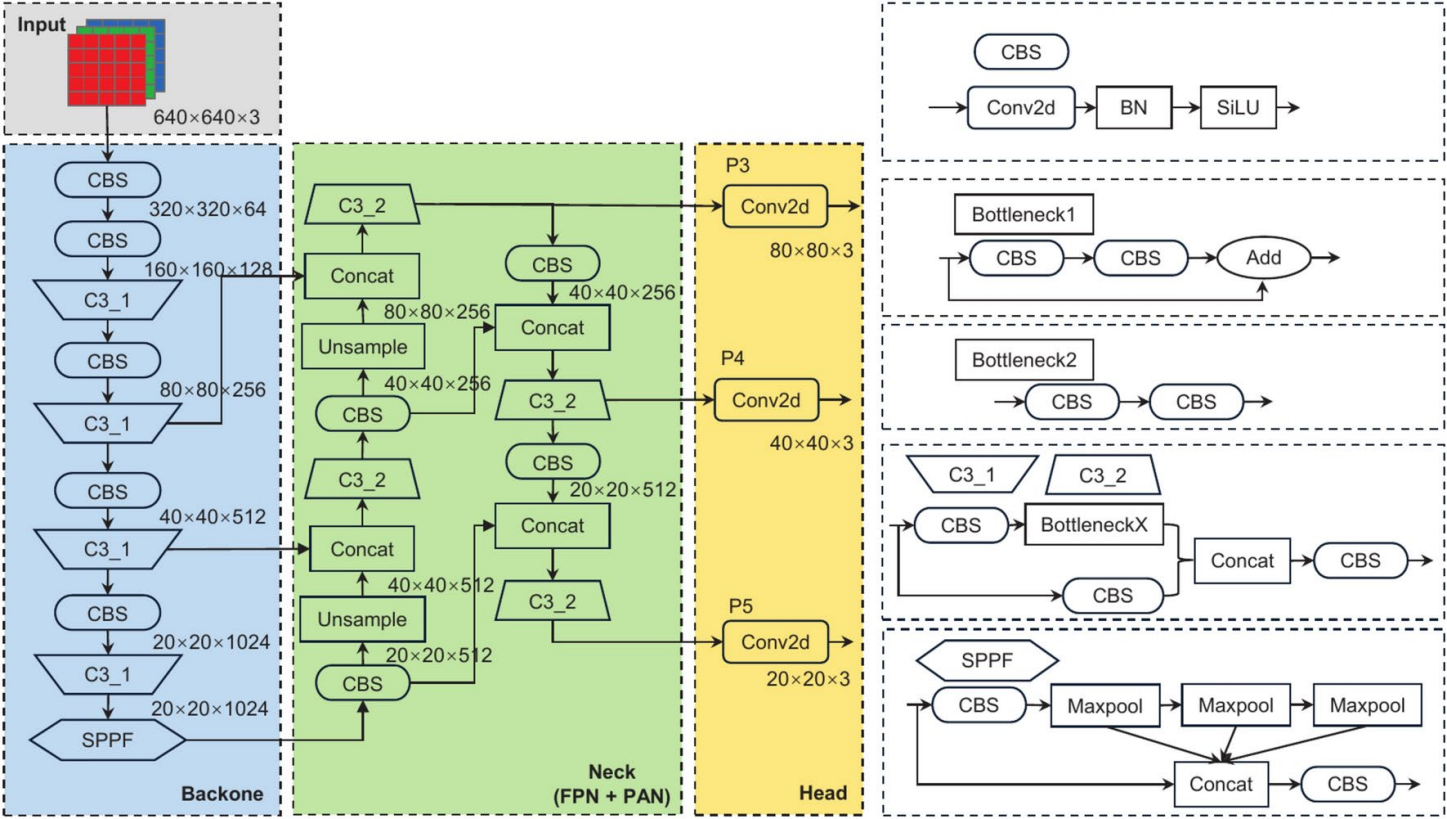
Network structure of YOLOv5.

As illustrated in Fig. 1, YOLOv5 consists of four main components: the input, backbone, neck, and head. The input incorporates adaptive anchor box computation based on the k-means algorithm, mosaic data augmentation, and adaptive image scaling to preprocess the training dataset, enhancing training efficiency. Based on the Darknet53 framework, the backbone integrates the C3 module, Spatial Pyramid Pooling-Fast (SPPF) module, and Convolutional Block Attention (CBS) to effectively extract target features from the input image. The neck combines the Feature Pyramid Network (FPN) and Path Aggregation Network (PAN) to merge feature maps at different scales, effectively integrating rich semantic and spatial information. Finally, the head performs convolutional operations on the outputs of the feature fusion network to predict the locations and categories of the detected objects.

**Fig. 2:**
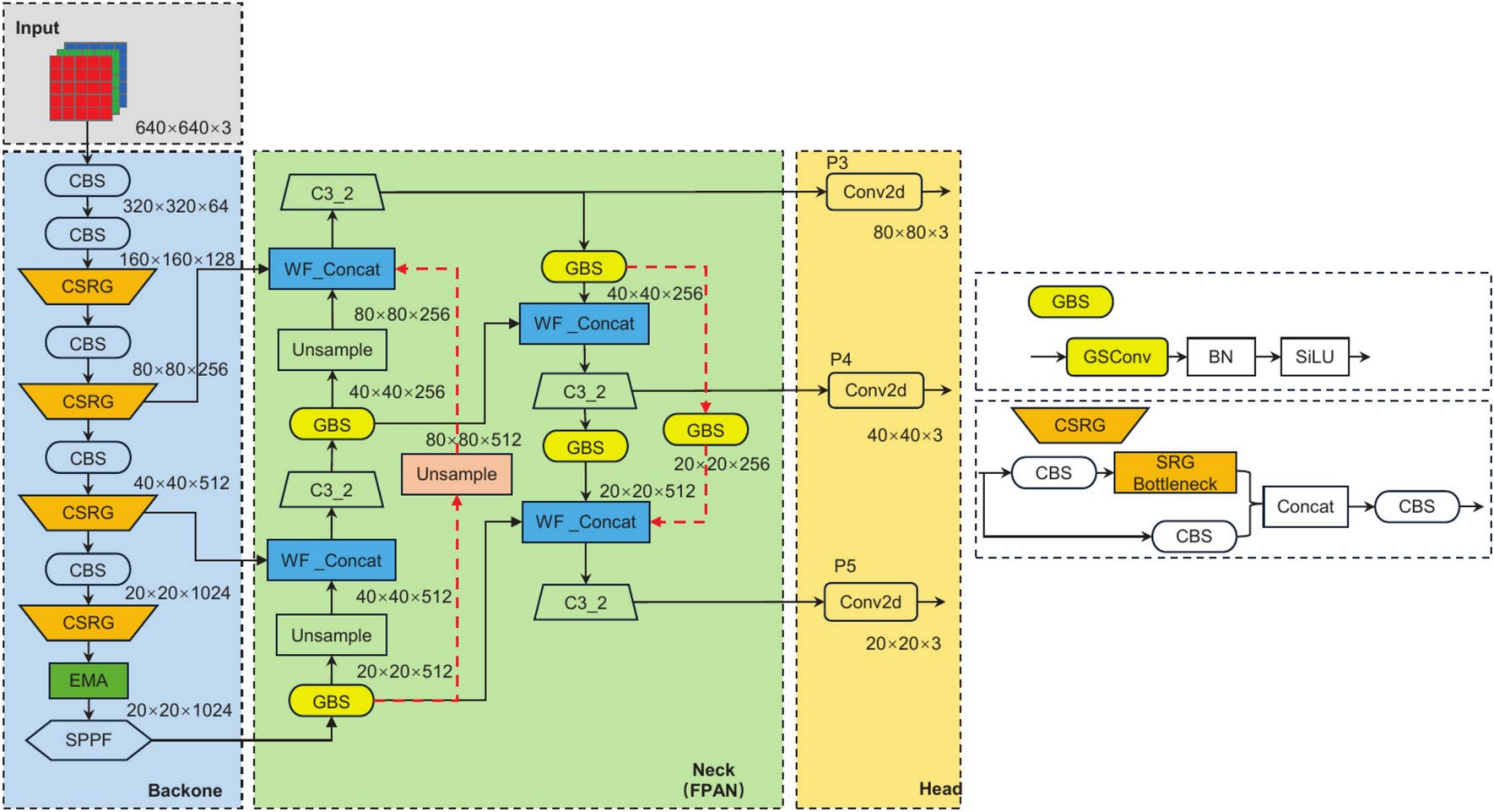
Network structure of LFD-YOLO.

## LFD-YOLO network structure design

The network structure of LFD-YOLO is shown in Fig. 2, where the colored boxes and red dashed lines highlight the key innovations of this research. First, we propose the CSRG feature extraction module, which reduces computational complexity while enhancing feature extraction capabilities. The introduction of the EMA module mitigates the impact of environmental noise, thereby improving the ability of the model to capture human posture features. Additionally, we optimize the feature fusion network by adopting the WFPN structure and replacing standard convolutions with GSConv, which effectively captures contextual information from multi-scale feature maps while reducing computational overhead. Furthermore, we design the Inner-WIoU loss function to accelerate model convergence and enhance the ability to fit bounding boxes under varying human scale changes. Overall, the LFD-YOLO model, through these improvements, reduces computational complexity and more accurately captures the intricate variations in human movement, thus enhancing the accuracy and stability of fall detection.

## Improvements of backbone

### CSRG lightweight module for feature extraction

#### Ghost convolution replaces standard convolution

**Fig. 3:**
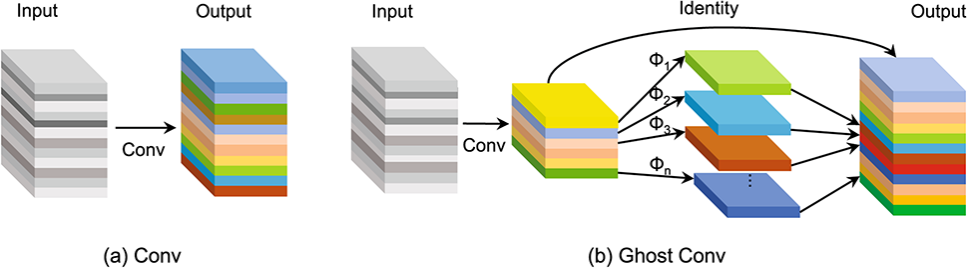
Structure of Conv and Ghost Conv.

Traditional standard convolution operations often generate redundant features when processing dynamic human motions in fall detection tasks, resulting in increased computational costs. To address this challenge, we proposes using Ghost convolution^26^ to replace the standard convolution in the feature extraction module. Ghost convolution aims to generate more informative features with fewer computations by creating and filtering features, thereby reducing redundant calculations and preserving only the useful features for the output. As shown in Fig. 3, Ghost convolution first uses a $1 \times 1$ convolution to compress the channel dimension of the input feature map, generating the feature map M. Next, each channel of M undergoes a convolution operation to extract more refined features. Finally, a linear transformation is applied to the convolved feature map, combined with the intermediate feature map M to produce the final output feature map.

Assuming that the shape of the input feature map is $(H_{in}, W_{in}, C_{in})$, the convolution kernel shape of standard convolution is $(K, K, C_{M})$, and the shape of the intermediate feature map, M, after compression by $1 \times 1$ convolution is $(H_{M}, W_{M}, C_{M})$ $(C_{M} < C_{in})$, the output feature map is $(H_{out}, W_{out}, C_{out})$. The number of parameters and calculations for standard convolution and Ghost convolution can be calculated as follows:

*Conv:*

1 $$\begin{aligned} Parameter= & K \times K \times C_{in} \times C_{out} \end{aligned}$$

2 $$\begin{aligned} Calculation= & \left[ (K \times K \times C_{in}) + (K \times K \times C_{in} - 1)\right] \times H_{out} \times W_{out} \times C_{out} \end{aligned}$$

*Ghost Conv:*

3 $$\begin{aligned} Parameter= & K \times K \times C_{in} \times C_{M} + 1 \times 1 \times \frac{C_{M}}{C_{M}} \times \frac{C_{out}}{C_{M}} \times C_{M} \end{aligned}$$

4 $$\begin{aligned} Calculation & = \left[ {(K \times K \times C_{{in}} ) + (K \times K \times C_{{in}} - 1)} \right] \times H_{M} \times W_{M} \times C_{M} \\ & \quad + \left[ {\left( {1 \times 1 \times \frac{{C_{M} }}{{C_{M} }}} \right) + \left( {1 \times 1 \times \frac{{C_{M} }}{{C_{M} }} - 1} \right)} \right] \times H_{{out}} \times W_{{out}} \times \frac{{C_{{out}} }}{{C_{M} }} \times C_{M} \\ \end{aligned}$$

Compared to standard convolution in Table 1, the number of parameters and calculations of Ghost convolution is reduced. $(H_{in} = H_{M} = H_{out} = H, W_{in} = W_{M} = W_{out} = W,C_{in} = C_{out} = 2C_{M} = 2C.)$

**Table 1:** Computational complexity of Conv and Ghost Conv.

Type	Parameter	Calculation
Conv	$4K^2C^2$	$8K^2HWC^2$
Ghost Conv	$2C + 2K^2C^2$	$4HWC + 4K^2HWC^2$

#### SRG bottleneck for improving feature expression ability

**Fig. 4:**
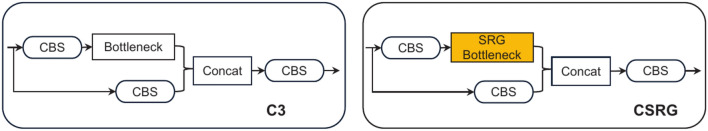
Structure of C3 and CSRG modules.

As shown in Fig. 4, the core component of the feature extraction network is the C3 module. This module relies on local feature learning but fails to fully exploit the contextual information from feature maps at different scales. Consequently, when capturing dynamic changes in human body features, this limitation may result in the loss of certain feature map information, reducing the efficiency of feature extraction. To address these issues, this research proposes replacing the C3 module’s bottleneck with an SRG bottleneck, as illustrated in Fig. 4. We reduce computational complexity by improving the module structure during both the training and inference phases while effectively minimizing information loss during the feature extraction process. This approach provides richer feature map information, enabling more efficient extraction of human pose features.

**Fig. 5:**
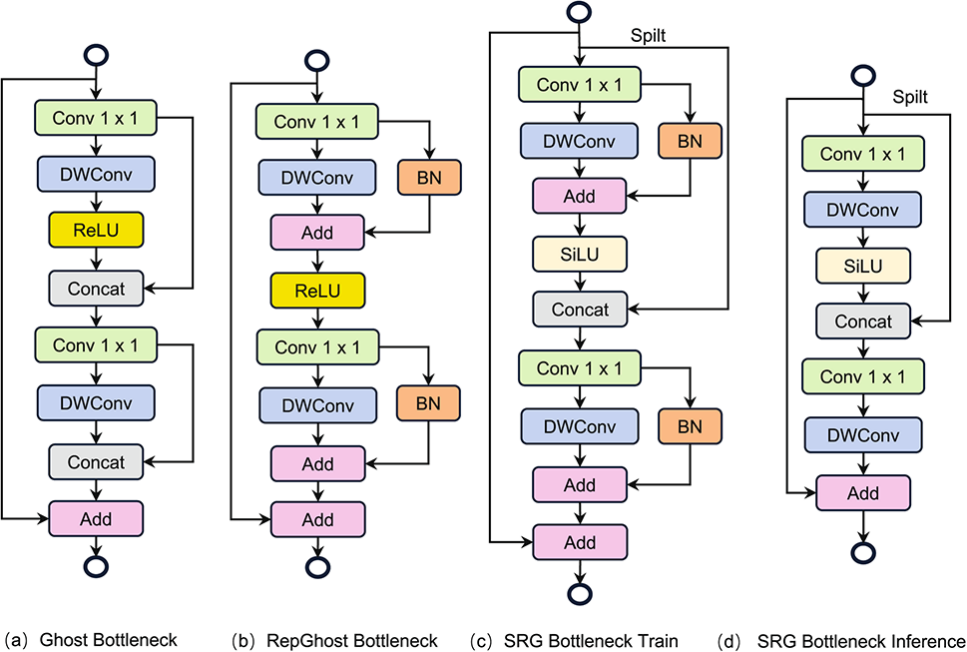
Structure of different feature extraction modules.

Figure 5 illustrates the structure of the feature extraction module before and after improvement. The traditional module consists of a series of convolutional operations. The Ghost bottleneck, constructed by stacking lightweight Ghost convolutions, offers a lower computational complexity. Building upon the Ghost bottleneck, the RepGhost bottleneck^27^ replaces the Concat operation with an Add operation, effectively reducing information redundancy during the concatenation process. Additionally, batch normalization (BN) layers are incorporated to enhance training stability. The SRG bottleneck, designed in this research, further optimizes the above structure by adjusting both the training and inference phase module structures. During the training phase, the SRG bottleneck retains the design of the RepGhost bottleneck while introducing a Split branch at the input stage. This modification reduces information loss during the feature map transmission process and provides richer feature information. Moreover, the activation function is replaced from the Rectified Linear Unit (ReLU)^28^ to the Sigmoid-weighted Linear Unit (SiLU)^29^. The SiLU demonstrates smoother behavior for lower input values and maintains higher gradients for higher input values, effectively mitigating issues such as gradient vanishing and overfitting, thereby improving training stability.

In the inference phase, we employs structural reparameterization^30,31^ based on the existing structure. This technique optimizes the model structure and improves inference efficiency by redesigning the module structure and altering the model’s computation process. Figure 5d shows that the convolutional layer and the BN layer are fused into an equivalent convolutional layer. This design effectively reduces inference time without compromising the model’s detection performance.

### EMA module for enhancing human pose attention

In fall detection scenarios, irrelevant environmental factors and human occlusions often disrupt the detection process, leading to missed or false detections. Traditional channel and spatial attention mechanisms^32^ typically compute attention weights across the entire feature map, increasing computational overhead and capturing only local dependencies, failing to recognize long-range dependencies effectively. As a result, the model’s detection accuracy deteriorates in the presence of external interference. To address this challenge, this research introduces the EMA attention mechanism. Since fall movements involve changes at different spatial scales, the EMA module^33^ fuses features from multiple scales through parallel branches. It employs an information aggregation strategy to capture long-range and short-range dependencies between feature maps, thereby reducing the impact of environmental disturbances and improvint the model’s focus on human posture.

**Fig. 6:**
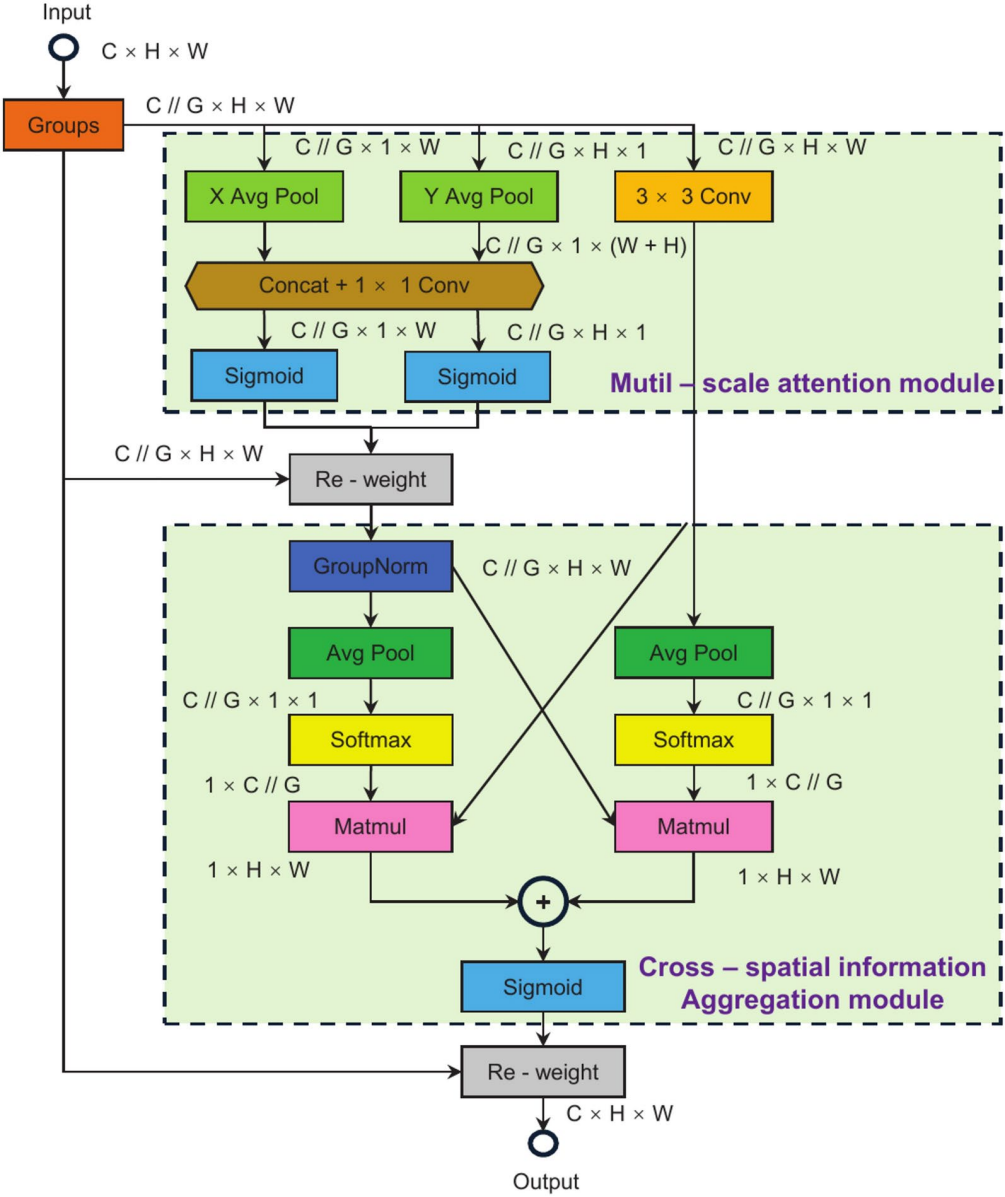
Structure of EMA module.

The EMA attention mechanism effectively integrates information from both the channel and spatial dimensions through parallel branches. First, the module adjusts part of the channel dimension to the batch dimension. It divides the channels into multiple sub-feature groups, ensuring a balanced distribution of spatial semantic features within each group and optimizing the channel weight distribution. In addition, the EMA attention mechanism introduces a cross-spatial information aggregation strategy. Through cross-dimensional interactions between parallel branches, it utilizes 2D global average pooling to encode global spatial information from the input feature map, capturing both long-range and short-range dependencies between feature maps. This approach enhances the capability of features to represent information across different scales. Figure 6 illustrates the structure of the EMA module. By incorporating a weighted average of historical human motion information, the EMA module captures the dependencies of feature information, reduces the impact of irrelevant information interference, and enhances the model’s focus on human detail features. This results in improved detection performance in cases of human occlusion, thereby enhancing detection accuracy and stability.

## Improvements of neck

### WFPN structure with weighted fusion strategy

The feature fusion network of YOLOv5s employs the FPN^34^ + PAN^35^ structure. This structure primarily relies on the fusion of adjacent layer feature maps, neglecting the information flow between non-adjacent layers, which leads to the loss of some information during the feature fusion process^36^. Furthermore, due to the differences in the information contained within feature maps of different scales, feature fusion through concatenation may result in information loss or redundant computations. To address these issues, we proposes an improved WFPN structure. This structure introduces cross-layer connections to fully capture the information flow between non-adjacent layer feature maps, thereby reducing the information loss during the transfer process. A weighted fusion strategy is also introduced to optimize the integration of feature maps at different scales, efficiently combining deep-level semantic information with shallow-level positional information, thus enhancing the expression ability of multi-scale feature maps.

**Fig. 7:**
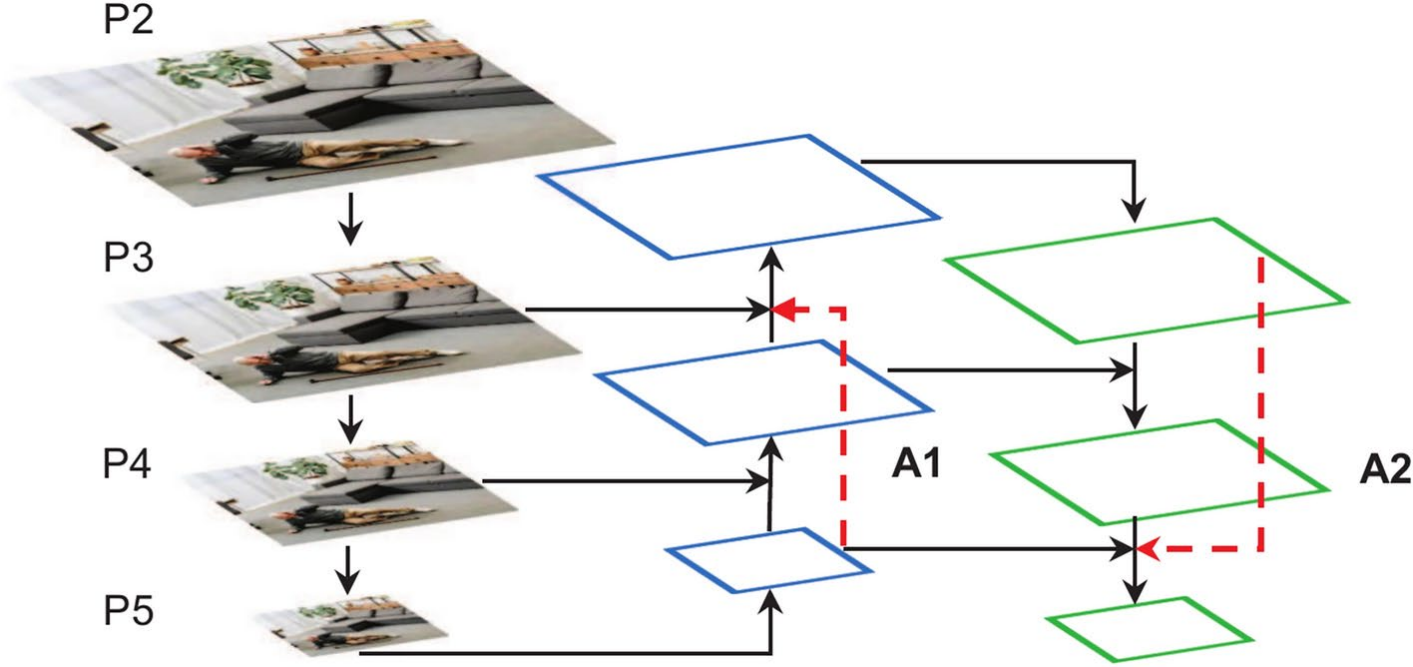
Structure of WFPN.

As shown by the red dashed lines in Fig. 7, two new cross-layer connections, A1 and A2, are added to the original structure. Since feature maps from different layers have varying spatial resolutions, resolution alignment is required during cross-layer fusion through upsampling or downsampling operations for effective connectivity. Figure 2 visually illustrates the spatial resolution changes of each feature map during the alignment and fusion process. Specifically, the A1 connection is located in the bottom-up path of the FPN, where deep-level feature maps are upsampled to increase their spatial resolution and fused with shallow-level feature maps from non-adjacent layers. The A2 connection is located in the top-down path of the PAN, where shallow feature maps are downsampled via lightweight convolution operations to reduce spatial resolution, facilitating fusion with deep-level feature maps from non-adjacent layers. The design of these two cross-layer connections aims to strengthen the information exchange between non-adjacent layer feature maps, improving the information utilization efficiency of feature maps from different layers.

Due to the differences in the information contained within feature maps of different scales, the WFPN structure introduces a weighted fusion strategy^37^ during multi-scale feature map fusion. This strategy allows the network to dynamically adjust the contribution weights of the feature maps to be fused based on their relative importance, thereby enhancing the information representation capability of feature maps at different scales. The mathematical expression for the weighted fusion strategy is as follows:

5 $$I_{{out}} = \sum\limits_{i} {\frac{{w_{i} }}{{\varepsilon + \sum\nolimits_{j} {w_{j} } }}} I_{{in}} {\text{ }}$$

In the equation, $I_{in}$ represents a series of input feature maps aligned in resolution to match the output feature dimensions. $I_{out}$ is the output feature map obtained by fusing the input feature maps according to their contribution weights. $w_{i}$ denotes the contribution weight assigned to feature maps at different levels, which is learned through network training and constrained within the range of 0 to 1. $\varepsilon$ is a small constant added to ensure computational stability. The weighted fusion strategy aims to allow the network to dynamically adjust the importance of information from feature maps at different scales during training, thus enabling efficient multi-scale feature map fusion without introducing additional computational overhead.

### GSConv with Low computational complexity

**Fig. 8:**
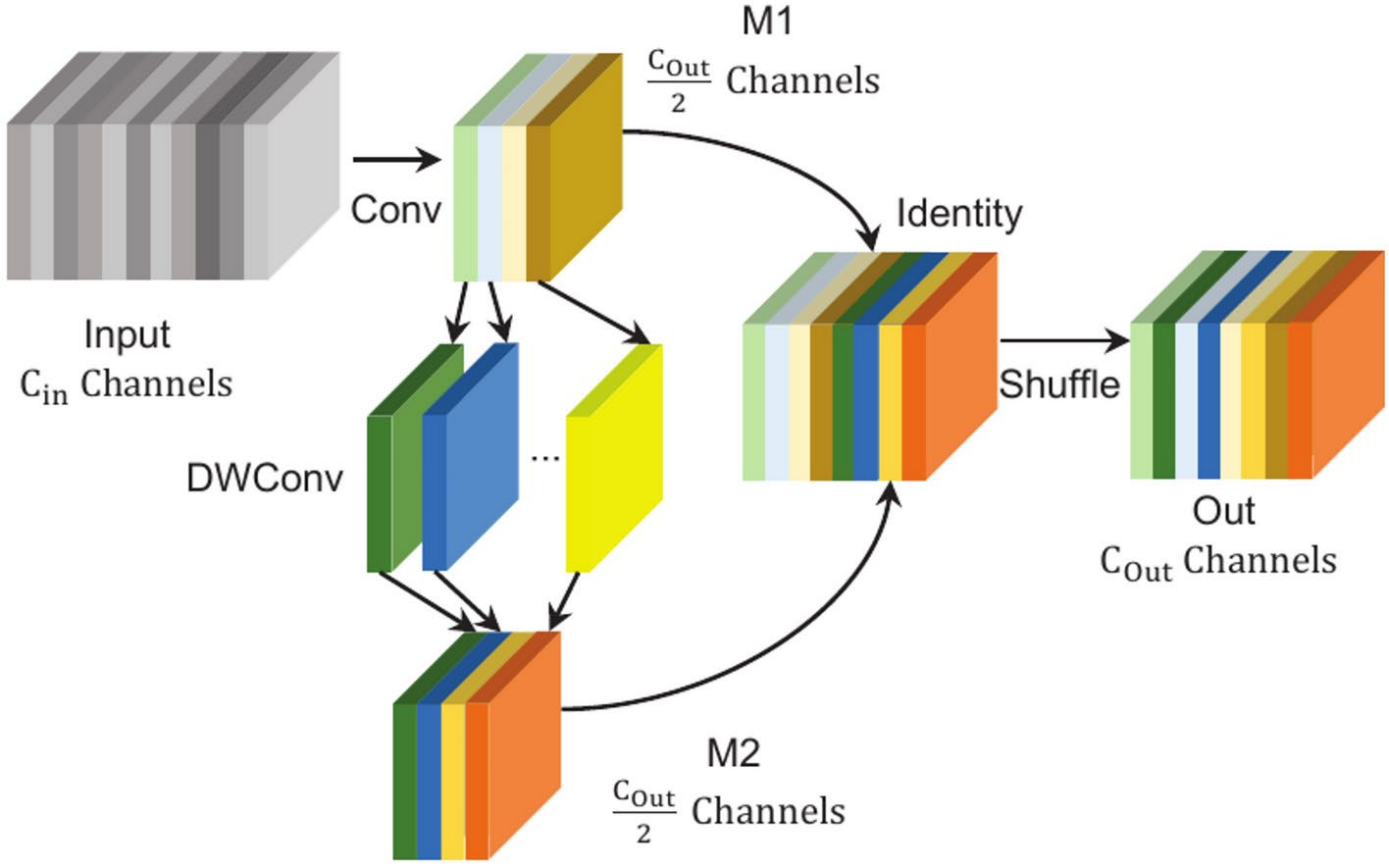
Structure of GSConv.

Given the requirements for model lightweight design and real-time performance in fall detection tasks, as well as the high computational complexity introduced by standard convolutions, this research replaces the standard convolutions in the feature fusion network with GSConv^38^. The GSConv aims to reduce computational complexity and enhance information exchange between feature channels. As shown in Fig. 8, GSConv executes a standard convolution operation on the input feature map, generating feature map M1. Then, each channel undergoes a convolution operation to produce the feature map M2. Next, M1 and M2 are concatenated and rearranged through a shuffle operation, allowing the feature information from M1 to penetrate every part of M2. This process enhances the information exchange between channels while significantly reducing the computational cost.

It should be noted that GSConv is only used in the feature fusion network. If applied across all stages of the model, while it could enhance the nonlinear expressive capacity, it may also increase the depth of the network layers. This could hinder the effective transmission of spatial and semantic information in the feature maps and significantly increase model inference time. Assuming that the input feature map has a shape of $(H_{in}, W_{in}, C_{in})$, with the convolution kernel size involved in the convolution being $(K, K, C_{M})$. Following compression by standard convolution, the shape of the feature map is $(H_{M}, W_{M}, C_{M})$ $(C_{M} < C_{in})$. The shape of output feature map is $(H_{out}, W_{out}, C_{out})$. The number of parameters and calculation of Conv and GSconv can be obtained from Eqs. (1)–(4) in the same way. The comparison in Table 2 shows that replacing standard convolution with GSConv reduces the model’s computational complexity. ($H_{in} = H_{M} = H_{out} =H, W_{in} = W_{M} = W_{out} = W, C_{in} = C_{out} = 4C_{M} = 4C$, the shuffle operation does not increase additional calculation loss.)

**Table 2:** Computational complexity of Conv and GSConv.

Type	Parameter	Calculation
Conv	$8K^2C^2$	$16K^2 HWC^2$
GSConv	$2C + 4K^2C^2$	$4HWC + 8K^2HWC^2$

## Improvement of loss function

### Inner-WIoU loss function improves model convergence and generalization

The loss function is employed to quantify the deviation between the model’s predictions and the ground truth, thereby guiding the adjustment of network weights. Consequently, the choice of loss function significantly impacts the detection performance of the model. In YOLOv5, the confidence loss and classification loss utilize binary cross-entropy to assess the accuracy of object category detection and its location. The bounding box regression loss adopts the Complete Intersection over Union (CIoU) loss function^39^ to measure the overlap between the predicted and ground truth bounding boxes. Although the CIoU loss function improves localization accuracy by incorporating a shape similarity metric, it may lead to potential issues in fall detection scenarios. Specifically, due to significant variations in the human body scale, the model may overemphasize the shape of bounding boxes during training, neglecting the optimization of center point localization accuracy. This could hinder the model’s training process. To address this issue, we propose the Inner-WIoU loss function, which combines the advantages of both WIoU (Weighted Intersection over Union) and Inner-IoU loss functions. This loss function introduces a gradient gain allocation strategy with a dynamic monotonic focusing mechanism, optimizing the center point deviation between the predicted and ground truth bounding boxes while reducing the impact of extreme samples. Additionally, auxiliary bounding boxes of varying scales are generated based on the predicted and ground truth boxes, using a scaling factor to accommodate the variation in the human motion scale. This approach accelerates model convergence and enhances its generalization ability.

The WIoU loss function^40^ introduces a dynamic non-monotonic focusing mechanism, using “outlier” values” to replace the traditional IoU for evaluating anchor box quality. This effectively mitigates the adverse gradient effects caused by extreme samples, enabling the model to focus more on high-quality samples, thereby enhancing the training process. Additionally, a center point distance attention mechanism is designed to optimize the center point deviation between the predicted and ground truth bounding boxes. The loss function is computed as follows:

6 $$L_{WIoU_{v1}} = R_{WIoU} L_{IoU} \sqrt{a^2 + b^2}$$

7 $$R_{WIoU} = \exp \left( \frac{(x-x_{gt})^2 + (y-y_{gt})^2}{(w_g^2 + H_g^2)^*}\right) \in [1,e]$$

8 $$L_{IoU}= 1 - IoU, L_{IoU} \in [0,1]$$

Among them, $x,y,x_{gt},y_{gt}$ represent the coordinates of the center points of the predicted and ground truth bounding boxes, respectively. At the same time, $W_{g}, H_{g}$ denote the width and height of the minimum bounding rectangle that encompasses both the predicted and ground truth boxes. The term $R_{WIoU}$ in the formula emphasizes the $L_{IoU}$ of normal-quality anchor boxes during the loss calculation. WIoU loss function v3 builds upon v1 by introducing a gradient gain allocation strategy with a dynamic non-monotonic focusing mechanism, defined through the outlier parameter $\beta$. The formula for calculation is as follows:

9 $$\beta= \frac{L_{IoU}^{*}}{L_{IoU}} \in [0, +\infty )$$

10 $$L_{WIoU_{v3}}= r L_{WIoU_{v1}}, r = \frac{\beta }{\delta \alpha ^{\beta -\delta }}$$

In these formulas, $L_{IoU}^{*}$ denotes the dynamically updated mean normalization factor for $L_{IoU}$, *r*, while *r* represents the gradient gain. The parameters $\alpha , \delta$ are hyperparameters that control the dispersion of the anchor boxes. When $\beta$ deviates from the normal range, it indicates that the anchor box quality is extreme. By assigning a smaller gradient gain *r*, the model can focus the bounding box regression more effectively on anchor boxes of normal quality.

Although the WIoU loss function theoretically optimizes the bounding box regression loss, it struggles to adapt to environmental variations and the diversity of human postures in practical fall detection scenarios, leading to insufficient model generalization. To address this challenge, we introduces the Inner-IoU loss function^41^. Based on the predicted and ground truth bounding boxes, this function generates auxiliary bounding boxes at different scales using a scaling factor. It calculates the bounding box regression loss for samples of varying quality, thereby accommodating changes in human scale. This approach accelerates model convergence and enhances its generalization ability.

11 $$L_{Inner-IoU}= 1 - IoU^{Inner}, L_{Inner-IoU} \in [0,1]$$

12 $$L_{Inner-WIoU}= L_{WIoU_{v3}} + IoU - IoU^{Inner}$$

**Fig. 9:**
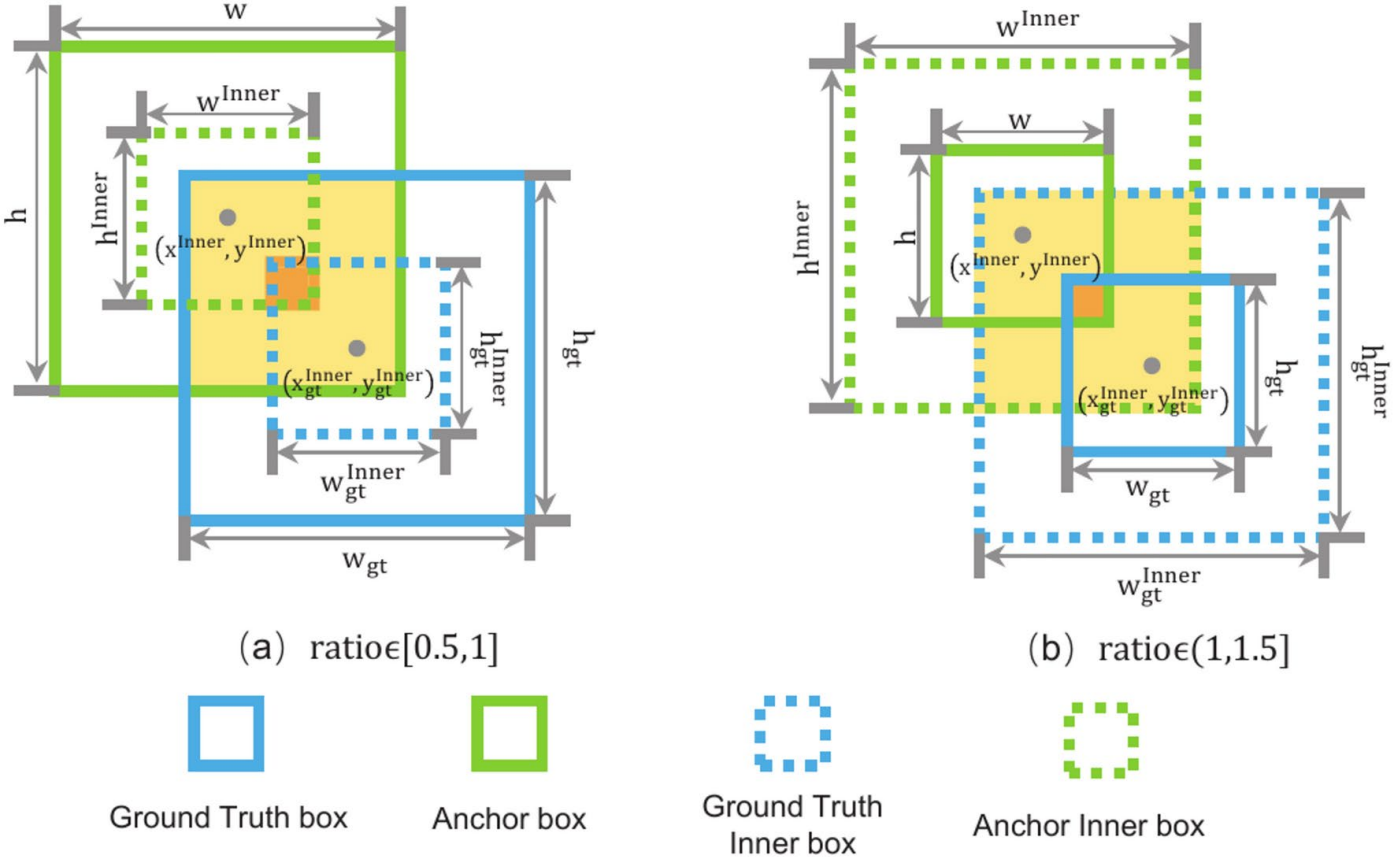
Inner boxes of different scales.

Figure 9 shows that the overlap between the auxiliary bounding boxes generated using different scaling factors and the ground truth bounding boxes. Since the auxiliary bounding boxes only differ in scale from the actual bounding boxes, their loss function is calculated similarly, as shown in Eq. (11). During the regression process, the trend in the IoU values of the auxiliary bounding boxes aligns with that of the actual bounding boxes, reflecting the quality of the actual bounding box regression. The $IoU^{Inner}$ represents the intersection over union between the predicted and ground truth auxiliary boxes, and $L_{Inner-IoU}$ can be computed using the auxiliary boxes. When the scaling factor is below 1, the auxiliary bounding box is smaller than the actual bounding box, reducing the effective regression range compared to $L_{IoU}$. However, its gradient magnitude is greater than that of $L_{IoU}$, accelerating the regression of high-quality samples. Conversely, when the scaling factor exceeds 1, the larger auxiliary bounding boxes expand the effective regression range, enhancing the regression gains for low-quality samples. Based on the Inner-WIoU loss function, further integration with WIoU v3 results in the Inner-WIoU loss function, the calculation of which is shown in Eq. (12). This loss function optimizes the bounding box regression process by increasing the focus on high-quality samples. Moreover, the scaling factor generates auxiliary bounding boxes at different scales, thus accelerating model convergence and improving its generalization ability.

## Experiment

### Datasets for fall detection

**Fig. 10:**
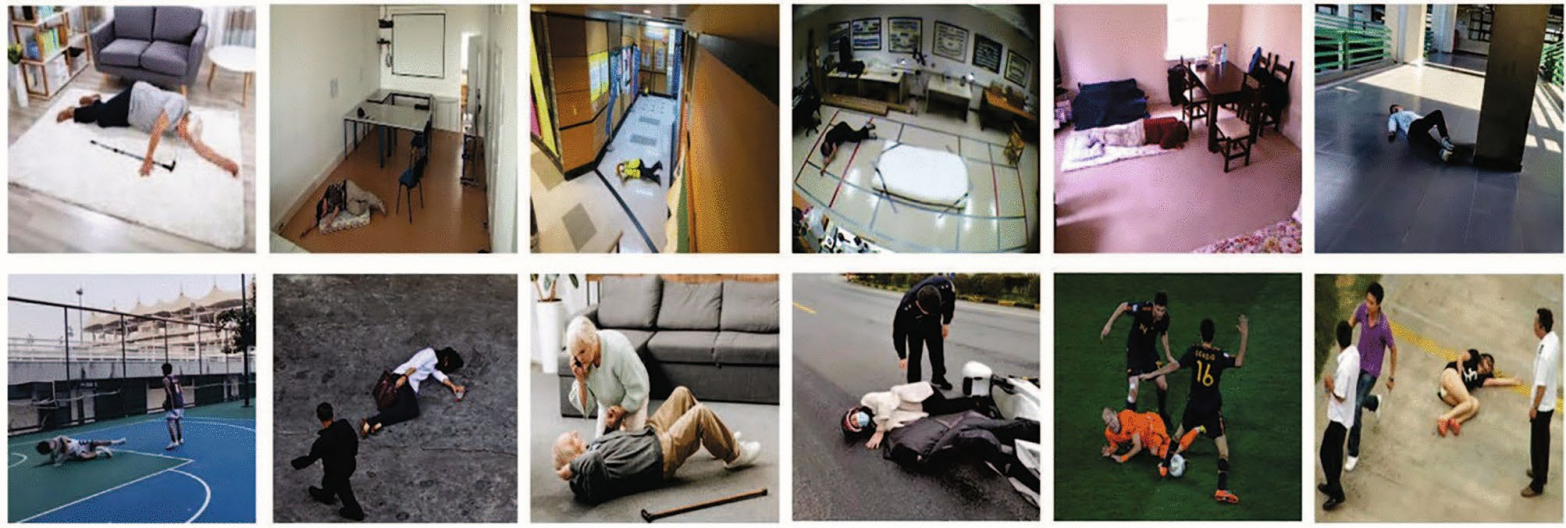
Partial dataset images.

In the field of object detection, the quality of datasets significantly influences the performance and generalization ability of deep learning algorithms. Existing publicly available fall detection datasets often feature limited scenarios and small sample sizes, which may not fully capture the complexity of real-world environments. Therefore, this study utilizes the publicly available FPID dataset^42^ and introduces a PFDD dataset for model training and evaluation. The FPID dataset consists of 8416 images depicting daily life scenarios, including normal activities and falls. The PFDD dataset, compiled from publicly available online^43^ and volunteer-submitted life images, contains 7859 carefully selected images. These images cover diverse scenarios, including different angles, lighting conditions, and human occlusion. All dataset images are uniformly annotated using the open-source tool LabelImg, with two categories: normal activities (“Person”) and falls (“Down”). A sample of the dataset images is shown in Fig. 10. Both datasets are split into training, validation, and test sets in a 7:1:2 ratio. To prevent model overfitting and enhance generalization, data augmentation techniques such as image flipping and Gaussian noise addition are applied to the training set. These measures increased the number of training samples and improved the dataset’s authenticity and diversity.

### Experimental environment

Table 3 presents the experimental setup. The model is trained for 300 iterations with input images resized to a resolution of $640 \times 640$ pixels. A batch size 16 is employed, and the initial learning rate is set to 0.01, which is gradually reduced using a cosine annealing schedule. The optimizer used is Stochastic Gradient Descent (SGD), with a weight decay coefficient of 0.0003 and a momentum value of 0.937 to control the learning rate.

**Table 3:** Experimental environment.

Environment	Configuration
Operating System	Ubuntu 20.04
CPU	Intel(R) Xeon(R) E5-2686 v4
GPU	NVIDIA GeForce RTX 3080-10G
Accelerated environment	CUDA11.8
Framework	Pytorch2.0.0
Language	Python3.8

### Indices for model evaluation

This research employs Precision (P), Recall (R), and Mean Average Precision (mAP) as metrics to evaluate model performance. Model size and computational resource requirements are also assessed using parameters and Giga Floating Point Operations Per Second (GFLOPS).

Precision reflects the proportion of correctly detected objects among all objects identified by the model. It is calculated using the following formula:

13 $$P = \frac{TP}{TP+FP}$$

Recall represents the proportion of true positive objects correctly identified by the model out of all actual positives. The formula is as follows:

14 $$R = \frac{TP}{TP+FN}$$

TP (True Positive) denotes the times that the model accurately detects a falling behavior as a fall. True Negative (TN) refers to the times the model correctly identifies a normal behavior. False Positive (FP) indicates when the model mistakenly identifies normal behavior as a fall. False Negative (FN) is when the model erroneously detects a falling behavior as usual.

The mAP represents the average of the precision scores averaged across all classes in the dataset. The formula is as follows:

15 $$mAP = \frac{1}{m} \sum _{i=1}^{m} AP_{i}$$

In the formula, m signifies the total count of categories within the samples, and $AP_{i}$ represents the average precision of the i-th class, obtained from the area enclosed by the Precision-Recall curve.

### Experimental results and analysis

To evaluate the performance of LFD-YOLO, we conduct ablation and comparison experiments on the FPID and PFDD datasets. The ablation experiment aims to assess the performance differences between YOLOv5s and its modified version, highlighting the specific contributions of each improvement to the overall model performance. The comparison experiment contrasts LFD-YOLO with current lightweight models, focusing on its relative advantages in terms of detection accuracy and computational complexity.

#### Ablation experiment

In the ablation experiments, several improvements to the LFD-YOLO model are validated. The experimental results are shown in Tables 4 and 5. Specifically, Model A represents the original YOLOv5s model. Model B incorporates the improved CSRG lightweight feature extraction module. As a result, the Parameters and GFLOPS decreased by 13.8% and 17.5%, respectively, while the mAP0.5 increased by 0.5% and 0.4%. These results indicate that the CSRG module, through its lightweight structure and split-branch design, reduces computational complexity while effectively minimizing feature map information loss, thus enhancing feature representation ability. Model C integrates the EMA attention mechanism. Without increasing computational resources, the mAP0.5 improved by 0.2%, demonstrating that this module, through multi-branch parallel computation, captures more human body information and enhances focus on human pose. Model D adopts the WFPN structure to optimize the feature fusion network. Although it incurs a slight increase in computational cost, the mAP0.5 improved by 0.5% and 0.6%. This suggests that the WFPN structure enhances information correlation between non-adjacent layers and dynamically adjusts the importance of feature maps, improving the efficiency of multi-level information fusion. Model E replaces standard convolution with GSConv. While detection performance slightly decreased, Parameters and GFLOPS are reduced by 7.4% and 6.0%, respectively. This confirms that GSConv effectively reduces computational complexity through its flexible convolution structure and shuffle operation. Model F substitutes the CIoU loss function with the Inner-WIoU loss function, leading to mAP0.5 improvements of 0.5% and 0.3%. With its gradient enhancement allocation strategy, this indicates that the Inner-WIoU loss function optimizes the center point offset between predicted and ground-truth boxes, thereby improving bounding box localization accuracy. Additionally, the loss function uses a scaling factor to generate auxiliary bounding boxes at different scales to fit human motion variations, thereby improving model generalization.

**Fig. 11:**
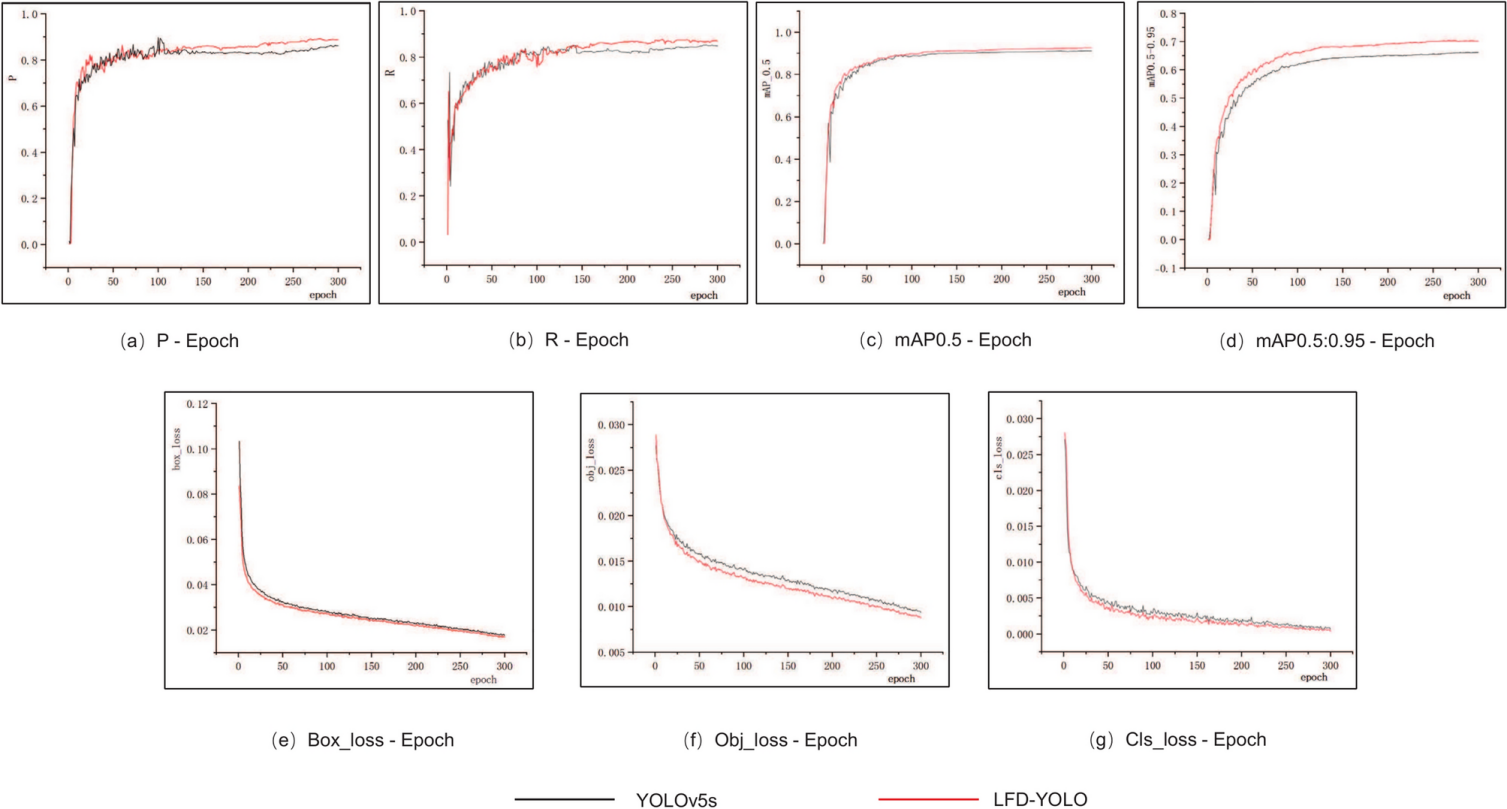
Performance curves of YOLOv5s and LFD-YOLO.

Overall, compared to YOLOv5s, LFD-YOLO reduces Parameters and GFLOPS by 19.2% and 21.3%, respectively, while mAP0.5 increases by 1.7% and 1.5%. The performance curves of both models on the PFDD dataset are shown in Fig. 11. The ablation experiment results demonstrate that the proposed improvements reduce the model’s computational complexity and effectively enhance detection accuracy, thereby validating the effectiveness of these improvements.

**Table 4:** Detection performance of improved modules on FPID dataset.

Model	CSRG	EMA	WFPN	GSConv	Inner-WIoU loss	P (%)	R (%)	mAP0.5 (%)	mAP0.5:0.95 (%)	Parameters (M)	GFLOPS
A	$\times$	$\times$	$\times$	$\times$	$\times$	80.9	75.7	83.0	46.1	7.02	16.0
B	$\checkmark$	$\times$	$\times$	$\times$	$\times$	81.5	76.2	83.5	46.6	6.05	13.2
C	$\checkmark$	$\checkmark$	$\times$	$\times$	$\times$	82.0	76.5	83.7	47.0	6.05	13.2
D	$\checkmark$	$\checkmark$	$\checkmark$	$\times$	$\times$	82.6	77.0	84.2	48.1	6.12	13.4
E	$\checkmark$	$\checkmark$	$\checkmark$	$\checkmark$	$\times$	82.4	76.7	83.9	47.5	5.67	12.6
F	$\checkmark$	$\checkmark$	$\checkmark$	$\checkmark$	$\checkmark$	83.1	77.3	84.6	48.8	5.67	12.6

**Table 5:** Detection performance of improved modules on PFDD dataset.

Model	CSRG	EMA	WFPN	GSConv	Inner-WIoU loss	P (%)	R (%)	mAP0.5 (%)	mAP0.5:0.95 (%)	Parameters (M)	GFLOPS
A	$\times$	$\times$	$\times$	$\times$	$\times$	85.2	84.8	91.2	66.1	7.02	16.0
B	$\checkmark$	$\times$	$\times$	$\times$	$\times$	85.9	85.6	91.6	67.7	6.05	13.2
C	$\checkmark$	$\checkmark$	$\times$	$\times$	$\times$	86.5	86.0	91.8	68.1	6.05	13.2
D	$\checkmark$	$\checkmark$	$\checkmark$	$\times$	$\times$	87.9	87.1	92.4	69.3	6.12	13.4
E	$\checkmark$	$\checkmark$	$\checkmark$	$\checkmark$	$\times$	87.6	86.8	92.1	68.8	5.67	12.6
F	$\checkmark$	$\checkmark$	$\checkmark$	$\checkmark$	$\checkmark$	88.4	87.5	92.7	70.2	5.67	12.6

#### Comparison experiment

The comparative experiments evaluate LFD-YOLO against current mainstream lightweight detection algorithms. The same dataset and experimental settings are used, with evaluation metrics including mAP, Parameters, and GFLOPS. The experimental results are shown in Tables 6 and 7. Compared to the two-stage model Faster R-CNN, LFD-YOLO achieved improvements of 6.6% and 4.2% in the mAP0.5 metric, respectively. When compared with the Transformer-based RT-DETR, LFD-YOLO improved mAP0.5 by 4.1% and 2.6%, respectively. For one-stage lightweight models, LFD-YOLO outperformed SSD, YOLOv5s, YOLOX-s, YOLOv7-tiny, and YOLOv8s by 4.9%, 1.7%, 1.4%, 2.6%, and 0.5%, respectively, in the mAP0.5 metric. Additionally, in the PFDD dataset, LFD-YOLO showed improvements of 3.4%, 1.5%, 1.1%, 2.2%, and 0.3% compared to these models. Regarding model size and computational resource consumption, LFD-YOLO exhibited a smaller model size and lower computational demands than other lightweight models.

**Fig. 12:**
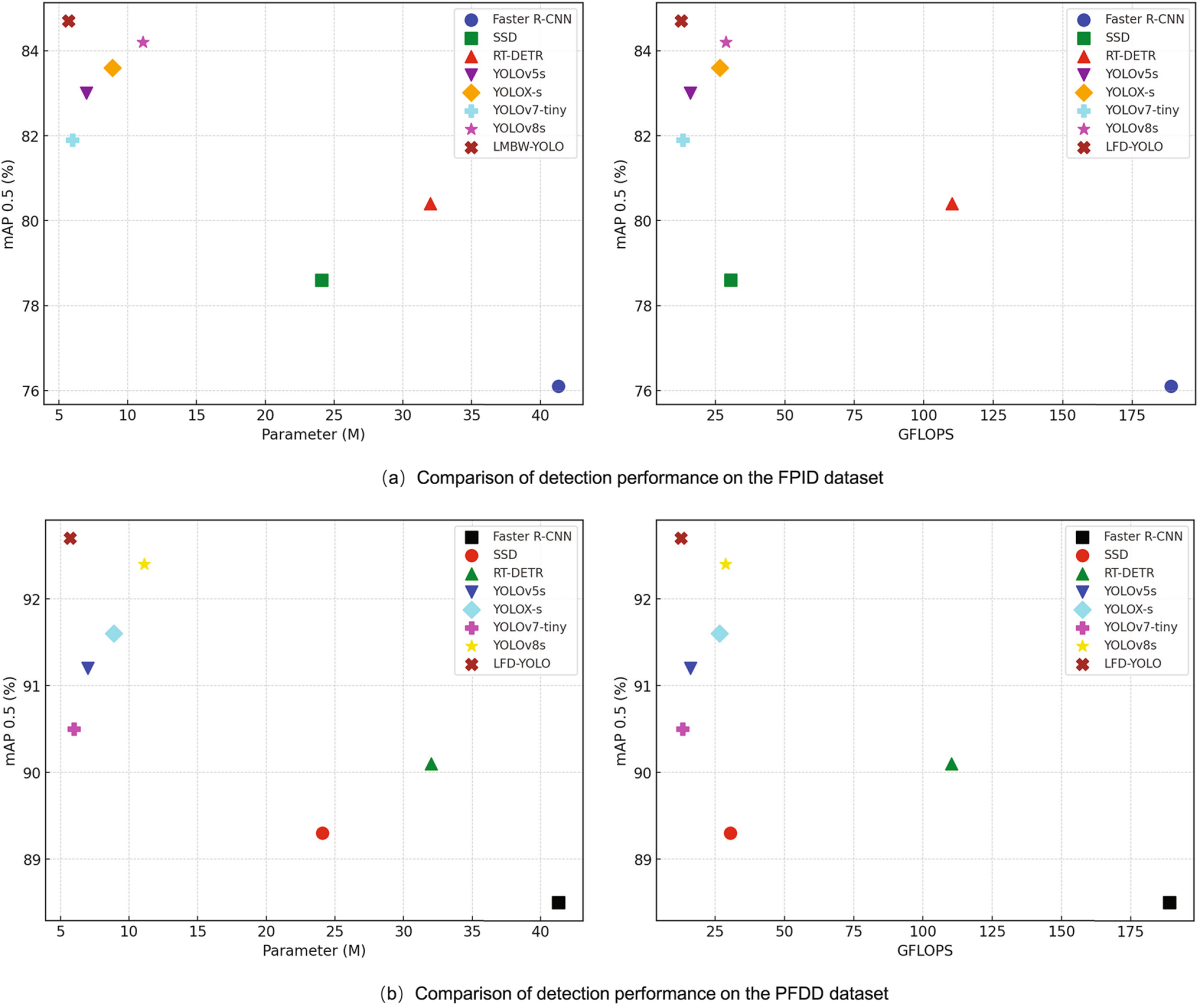
Comparison of detection performance across lightweight models.

Compared to the current advanced YOLOv8s lightweight model, LFD-YOLO reduced Parameters and GFLOPS by 48.6% and 56.1%, respectively, while improving mAP0.5 by 0.5% and 0.3%. Figure 12 shows detection performance across different lightweight models. The results of the comparative experiments demonstrate that LFD-YOLO strikes a better balance between detection performance and resource consumption, offering superior overall performance compared to current mainstream lightweight detection models.

**Table 6:** Detection performance of lightweight models on FPID dataset.

Model	mAP0.5 (%)	mAP0.5:0.95 (%)	Parameters (M)	GFLOPS
Faster R-CNN	76.1	37.5	41.3	189
SSD	78.6	39.7	24.1	30.5
RT-DETR	80.4	42.2	32.0	110.2
YOLOv5s	83.0	46.1	7.0	16.0
YOLOX-s	83.6	47.8	8.9	26.6
YOLOv7-tiny	81.9	45.6	6.0	13.2
YOLOv8s	84.2	48.5	11.1	28.7
LFD-YOLO	84.7	48.8	5.7	12.6

**Table 7:** Detection performance of lightweight models on PFDD dataset.

Model	mAP0.5 (%)	mAP0.5:0.95 (%)	Parameters (M)	GFLOPS
Faster R-CNN	88.5	60.9	41.3	189
SSD	89.3	62.3	24.1	30.5
RT-DETR	90.1	64.6	32.0	110.2
YOLOv5s	91.2	66.1	7.0	16.0
YOLOX-s	91.6	68.8	8.9	26.6
YOLOv7-tiny	90.5	65.2	6.0	13.2
YOLOv8s	92.4	70.4	11.1	28.7
LFD-YOLO	92.7	70.2	5.7	12.6

#### Scene testing and visualization

**Fig. 13:**
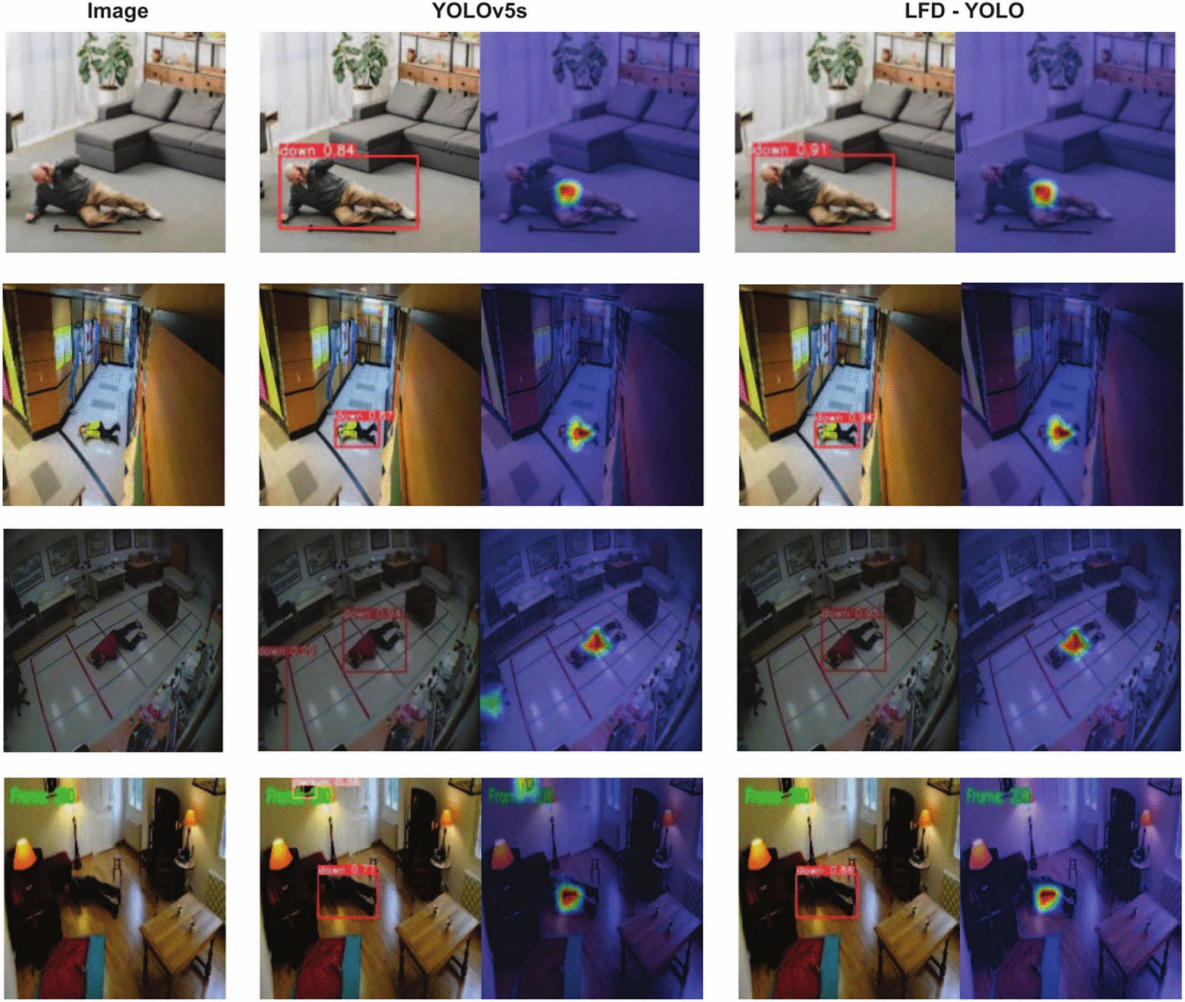
Detection results of different lighting conditions.

**Fig. 14:**
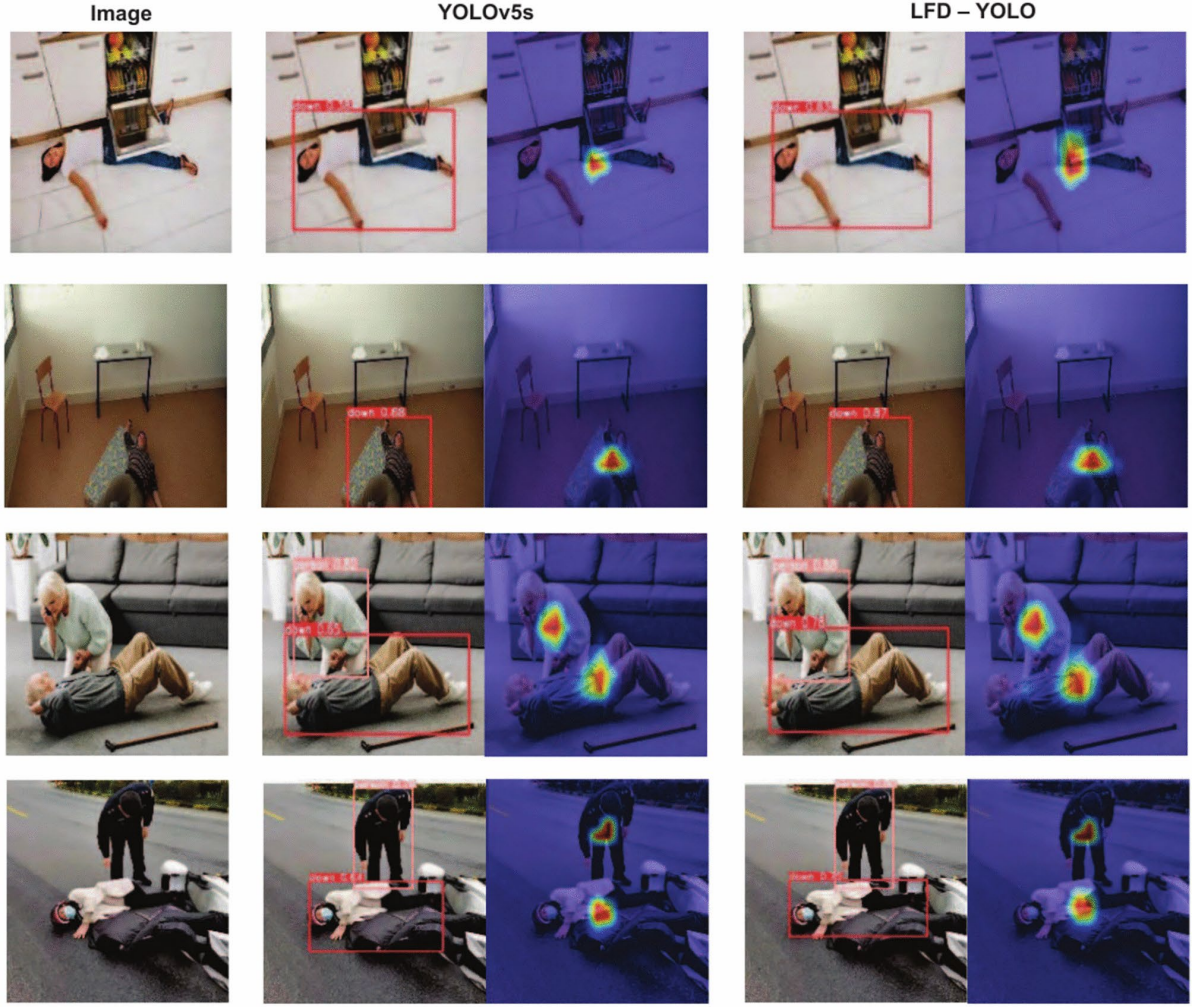
Detection results of human body occlusion.

This study selects publicly available images under varying lighting conditions and with human occlusion to compare the performance of YOLOv5s and LFD-YOLO in fall detection in real-world scenarios. The detection results are shown in Figs. 13 and 14. On the left side of the figures are the scene images, while the middle and right sections display the detection results and Grad-CAM visualizations for YOLOv5s and LFD-YOLO, respectively, revealing the performance differences between the two models under various conditions.

Figure 13 indicates that in low-light environments, YOLOv5s tend to misidentify objects in the surroundings as humans, leading to a reduction in detection accuracy. In contrast, LFD-YOLO consistently detects falls accurately across different lighting conditions. This result suggests that LFD-YOLO, through its improvements, effectively reduces the impact of environmental interference, enhances the representation of human pose features, and improves the model’s generalization ability. Figure 14 demonstrates that in cases of partial human occlusion, YOLOv5s struggles with reduced human information, resulting in a decrease in detection accuracy. On the other hand, LFD-YOLO maintains a high detection precision even with partial occlusion, indicating that the model’s improvements have increased its focus on human pose, allowing it to extract more human features and effectively mitigate the impact of occlusion.

Overall, a comparison of detection results across different scenarios shows that LFD-YOLO outperforms YOLOv5s in fall detection, exhibiting higher accuracy and stability while effectively reducing both missed detections and false positives. Therefore, the proposed improvements in this study reduce the computational complexity of LFD-YOLO and significantly enhance its detection performance and generalization ability, better meeting the requirements of fall detection tasks.

### Discussion and limitation

The LFD-YOLO model proposed in this study demonstrates excellent performance in fall detection tasks through a series of improvements. Experimental results illustrate that these enhancements effectively reduce computational complexity and improve detection accuracy. The ablation study results indicate that the CSRG lightweight feature extraction module reduces Parameters and GFLOPS by 13.8% and 17.5%, respectively, while increasing mAP. This suggests that the module enhances feature representation capabilities while reducing computational overhead. The EMA attention mechanism improves mAP0.5 by 0.2% without adding extra computational resources, indicating that it effectively strengthens attention to human pose and reduces the impact of environmental interference. The adoption of the WFPN structure, although introducing a slight computational cost, improves mAP0.5 by 0.5% and 0.6%, respectively. The optimized network reduces information loss during transmission and enhances multi-level feature maps’ fusion efficiency. Although using GSConv slightly reduces detection performance, it decreases Parameters and GFLOPS by 7.4% and 6.0%, lowering computational complexity. The Inner-WIoU loss function optimizes bounding box localization accuracy, further improving the generalization ability of the model. Overall, these improvements reduce computational complexity and enhance detection accuracy. It outperforms YOLOv5s in terms of performance and surpasses other mainstream lightweight detection models, such as YOLOv8, demonstrating its advantages in fall detection. Furthermore, LFD-YOLO maintains accurate fall detection under varying lighting conditions and human occlusion, further validating that the improvements enhance the expression of human pose features and the model’s generalization ability.

However, this study has some limitations. Firstly, while LFD-YOLO performs well on the FPID and PFDD datasets, complex backgrounds in real-world applications could impact model performance. Therefore, background modeling techniques could be introduced to reduce the interference of background variations. Furthermore, although LFD-YOLO performs well in fall detection, many human actions resemble fall behaviors, which may lead to false positives. Thus, the model can be extended to detect other human actions and more accurately distinguish similar movements, thereby enhancing its generalization ability. Overall, despite the achievements of LFD-YOLO in fall detection tasks, its performance in complex backgrounds and diverse human actions still requires further validation and optimization.

## Conclusion

To balance detection accuracy and computational complexity, this study proposes a lightweight fall detection model, LFD-YOLO, based on YOLOv5, incorporating a series of improvements. First, the CSRG feature extraction module is introduced, which employs a lightweight structure and adds a split-branch design to reduce computational overhead while providing richer feature map information. The EMA attention mechanism is also introduced, effectively capturing more human feature information through multi-branch parallel computation. Furthermore, a WFPN structure is designed to enhance the correlation between non-adjacent layer feature maps and dynamically adjust the relative importance of each layer’s feature map. This reduces information loss during the transmission process and improves the efficiency of multi-level feature map fusion. GSConv convolution replaces standard convolution, further reducing computational complexity. Moreover, the Inner-WIoU loss function improves the model’s localization accuracy and generalization ability.

Multiple experiments are conducted on the FPID and PFDD datasets. Compared to YOLOv5s, LFD-YOLO improved mAP0.5 by 1.7% and 1.5% while reducing Parameters and GFLOPS by 19.2% and 21.3%, respectively. Compared to the current advanced YOLOv8s model, LFD-YOLO reduced Parameters and GFLOPS by 48.6% and 56.1%, respectively, while improving mAP0.5 by 0.5% and 0.3%. The experimental results show that, through these improvements, LFD-YOLO reduces computational complexity and significantly enhances detection accuracy, better meeting the needs of fall detection tasks.

Although the detection performance of LFD-YOLO has improved, challenges remain in addressing complex background interference and distinguishing similar actions. Additionally, to facilitate deployment on low-cost edge devices, further research is needed on applying knowledge distillation and model pruning techniques. These methods can optimize the model structure and reduce computational resource requirements.

## Data Availability

The datasets generated during and/or analysed during the current study are available from the corresponding author on reasonable request.
